# Protective effect of cyclosporine A in the treatment of severe hydronephrosis in a rabbit renal pelvic perfusion model

**DOI:** 10.3906/sag-1901-193

**Published:** 2019-10-24

**Authors:** Ben-Zheng ZHOU, Da-Hu ZHANG, Wei-Min YU, Jin-zhuo NING

**Affiliations:** 1 Department of Urology, Xiangyang No.1 People’s Hospital, Hubei University of Medicine, Xiangyang, Hubei P.R. China; 2 Department of Urology, Renmin Hospital of Wuhan University, Wuhan, Hubei P.R. China

**Keywords:** Hydronephrosis, cyclosporine A, kidney injury, renal pelvic perfusion, renal protection

## Abstract

**Background/aim:**

Cyclosporine A (CsA), a traditional immunosuppressive compound, has been reported to specifically prevent ischemia reperfusion tissue injury via apoptosis pathway. This study aimed to explore the renoprotective effects of CsA on the kidneys of rabbits undergoing renal pelvic perfusion.

**Materials and methods:**

A total of 30 rabbits were randomly assigned into a control group (n = 6) and an experimental group (n = 24). The experimental group underwent a surgical procedure that induced severe hydronephrosis and was then stochastically divided into 4 groups (S1, S1’, S2, and S2’), consisting of 6 rabbits each. Groups S1 and S1’ were perfused with 20 mmHg of fluid, while groups S2 and S2’ were perfused with 60 mmHg of fluid. Administration to groups S1’ and S2’ was done intravenously, with CsA once a day for 1 week before perfusion. In the control group, after severe hydronephrosis was induced, a sham operation was performed in a second laparotomy. Acute kidney damage was evaluated using hematoxylin and eosin staining, in addition to analyzing the mitochondrial ultrastructure and mitochondrial membrane potential (MMP). The cytochrome C (CytC) and neutrophil gelatinase-associated lipocalin (NGAL) expression were examined immunohistochemically using Western blotting and reverse transcription-polymerase chain reaction.

**Results:**

It was found that the renal histopathological damage was ameliorated, mitochondrial vacuolization was lower, MMP was higher, and the CytC and NGAL contents were decreased after drug intervention (groups S1’ and S2’) when compared to the experimental groups (S1 and S2). Furthermore, there was no difference between drug intervention groups S1’ and S2’.

**Conclusion:**

These results suggest that CsA can attenuate renal damage from severe hydronephrosis induced by renal pelvic perfusion in rabbits. It plays a protective role in the acute kidney injury process, possibly through increased MMP and mitochondrial changes.

## 1. Introduction

With the development of minimally invasive technology, many types of ureteroscopy and percutaneous nephroscope lithotripsy have become routine procedures in surgical intervention for kidney stones, as they have several advantages, including reduced postsurgical pain, efficient stone clearance, shorter hospitalization time, and reduced scar formation than open surgery [1,2]. However, minimally invasive surgery in the kidney may not be minimally invasive within the kidney itself. Endourological operations need sufficient fluid perfusion to clearly flush out kidney stone fragments during these procedures, due to the limitations of the orifices [3]. These procedures can cause high intrapelvic pressure and pyelovenous backflow when the pressure increases to a certain extent, which could reduce renal arterial perfusion and lead to renal ischemic injury [4,5]. In addition, there is a certain degree of hydronephrosis in patients with upper urinary tract stones. A previous study demonstrated that a 60 mmHg renal pelvic perfusion substantially aggravated kidney damage in a rabbit model of hydronephrosis via mitochondrial injury [6]. Nevertheless, effective protection for hydronephrotic kidneys after renal pelvic perfusion has not yet been studied.

Cyclosporine A (CsA), known as an immunosuppressive compound, has been traditionally used to prevent and treat transplant rejection [7]. Recently, CsA has been thought to specifically prevent mitochondrial permeability transition pore (mPTP) opening and attenuate cell apoptosis by exerting cardioprotective effects in a reperfusion injury model [8]. Moreover, previous studies have shown that CsA protects against tissue ischemia-reperfusion injury in the brain [9], lung [10], and kidney [11] in vivo. However, whether CsA affects renal pelvic perfusion-induced hydronephrotic kidney injury in vivo is unknown. Although CsA is known as a nephrotoxic drug, a low dose of CsA was safe and effective in animal models, according to previous experimental studies [12]. 

Herein, it was speculated that severe hydronephrosis can cause renal parenchymal ischemic injury, based on rabbit models involving renal pelvic perfusion of 60 mmHg, as well as mitochondrial damage in renal tubular epithelial cells. Hence, the mPTP inhibitor CsA was chosen to pretreat large white rabbits in an experimental group in order to observe the renoprotective effects of CsA on kidneys undergoing pelvic perfusion.

## 2. Materials and methods

### 2.1. Animals and groups

A total of 30 adult New Zealand white rabbits (1.9–2.3 kg) were received from the Wuhan Institute of Biological Products Co., Ltd. (Wuhan, China). All of the procedures were approved by the Animal Experimental Ethics Committee of Wuhan University (Wuhan, Hubei, China). All of the rabbits were stochastically assigned into a control group (n = 6) and an experimental group (n = 24). Severe hydronephrosis was induced in the experimental group via surgery and the rabbits were then stochastically divided into 4 groups (S1, S1’, S2, and S2’), consisting of 6 rabbits each, after successful molding by B ultrasonic examination. Groups S1 and S1’ were perfused with 20 mmHg of fluid, while groups S2 and S2’ were perfused with 60 mmHg of fluid. Groups S1’ and S2’ were administered CsA intravenously once a day for 1 week before the perfusion. In the control group, after severe hydronephrosis was induced, a sham operation was performed in a second laparotomy. Acute kidney injuries were evaluated by hematoxylin and eosin (HE) staining, in addition to analyzing the mitochondrial ultrastructure and mitochondrial membrane potential (MMP). CytC expression was analyzed immunohistochemically and the NGAL expression was examined using Western blotting.

### 2.2. Surgical manipulation

Rabbits in the experimental and control groups underwent an operation to induce severe hydronephrosis according to a previous method [13]. The animals were anesthetized using pentobarbital [30 mg/kg, intravenously (i.v.)] via the marginal vein of the ear. They were fixed in a supine position. After a midline abdominal incision, the left lumbar vein, left ureter, and psoas muscle were exposed. The upper two-thirds of the ureter was covered by the psoas muscle, and the muscles were sutured over the ureter. Two weeks after surgery, B-ultrasonography was performed to evaluate hydronephrosis. Pelvic distention was 1.72 ± 0.38 cm, while the thickness of the parenchyma was 0.21 ± 0.03 cm. After hydronephrosis was confirmed, groups S1’ and S2’ were administered CsA (2.5 mg/kg, i.v.) once a day for 1 week. The dose of CsA used was safe and effective in animal models according to a previous study [12]. Next, the second laparotomy was performed. The left kidney was revealed, and subsequently, a size 4.5 needle was embedded approximately 0.4 cm into the parenchyma. After that, a pressure pump and physiological recorder were connected to maintain and detect the intrapelvic pressure. Before perfusion, the intrapelvic pressures were measured in the severely hydronephrotic kidneys and were found to be 16.35 ± 1.85 mmHg. Groups S1 and S2 were irrigated at 20 and 60 mmHg with warm isotonic saline for 8 min. The irrigation was then paused for 2 min. This procedure was carried out for 4 times. In the end, the sutured ureter was released, and the abdominal median incision was sutured. For the control group, the hydronephrotic kidney was only exposed without perfusion in the second laparotomy. The rabbits were euthanized by air injection 48 h after that, and then, the left renal tissue specimens were immediately separated to analyze the MMP and mitochondrial ultrastructure. Partial tissue specimens for the immunohistochemical analyses were embedded in paraffin, while the other kidney tissues were frozen at –80 °C until performing the Western blot analysis.

### 2.3. HE staining

After 10% formalin fixation, the renal tissues were processed routinely for paraffin embedding and cut into 5-μm slices. After general dewaxing and hydration, the slices were then stained with HE.

### 2.4. Kidney ultrastructure by transmission electron microscopy

Tissues were dissected and fixed with 2.5% glutaraldehyde, rinsed in 0.1 M phosphate buffer 3 times, postfixed in 1% buffered osmium tetroxide, and rinsed in 0.1 M phosphate buffer. Next, the tissues were dehydrated by incubating the tissue in graded alcohol, and the dehydrated tissue was put into epoxy resin. Thin tissue sections were cut using an ultramicrotome (LKB, Bromma, Kista, Sweden) and stained with uranyl acetate and lead citrate. A transmission electron microscope was used to observe any ultrastructural changes (H-600, Hitachi, Tokyo, Japan). Mitochondrial morphology changes were determined in images from 3–5 random fields in each group for the calculation of the percentage of vacuolar mitochondria.

### 2.5. MMP detection

MMP was detected using 5,5’,6,6’-tetrachloro-1,1’,3,3’tetraethylbenzimidazolycarbocyanine iodide (JC-1, Beyotime Institute of Biotechnology, Jiangsu, China). Kidney tissue was digested in a trypsin-ethylene-diamine-tetraacetic acid (EDTA) solution (Beyotime Institute of Biotechnology). Subsequently, the bovine serum was added to end digestion. Suspended cells were harvested and loaded with 1×JC-1 at 37 °C for 20 min, and then, the cells were washed and detected by flow cytometry (FACS Aria III, BD, New Jersey, US). When the MMP is at a low level, JC-1 exists mainly as a monomer that emits green fluorescence (excitation wavelength of 488 nm and emission wavelength of 530 nm). When the MMP is at a high level, JC-1 exists mainly as a polymer that emits red fluorescence (excitation wavelength of 525 nm and emission wavelength of 590 nm). For data presentation, the value of the MMP was expressed as the intensity ratio of red to green fluorescence.

### 2.6. Immunohistochemical staining

Briefly, the kidney tissues were paraffin-embedded, sectioned, fixed, and dehydrated. Endogenous enzymes were deactivated by applying 0.3% hydrogen peroxide for 10 min at 37 °C. Microwave antigen retrieval of the CytC and NGAL was performed on the rehydrated slides in a citric acid buffer solution and Tri/EDTA buffer solution for 15 min. Samples were incubated with a primary antibody against CytC and NGAL (Univ-Biotechnology, Shanghai, China) overnight at 4 °C. Next, the secondary antibodies were added, and the samples were incubated. After redying, purifying, dehydrating, and cleaning, the samples were observed under a microscope. The immunostaining scores were conducted according to the manufacturer’s recommendations.

### 2.7. Western blotting

The protein levels of the NGAL were detected using the Western blotting analysis. Briefly, the bicinchoninic acid method was used to measure protein concentrations, and the kidney proteins (10 µg) were separated by electrophoresis in 10% sodium dodecyl sulfate polyacrylamide gels. Proteins in the gels were transferred to nitrocellulose membranes. The membranes were then probed with antiNGAL polyclonal antibodies (1:500; Bioss Biological Technology Co., LTD, Beijing, China) and antiCytc monoclonal antibodies (1: 2000; Bioss Biological Technology Co.) for rabbit proteins. HRP-conjugated goat antirabbit IgG antibodies (Cell Signaling Technology, Shanghai, China) were applied as secondary antibodies (1: 10,000). The relevant protein bands were visualized by enhanced chemiluminescence reagents and analyzed with the Odyssey image analysis system. To determine an equal content of proteins for each sample, the membranes were reprobed with mouse anti-glyceraldehyde-3-phosphate dehydrogenase (GAPDH) polyclonal antibodies (1: 5000, Cell Signaling Technology). The result was expressed as the ratio of target protein to GAPDH.

### 2.8. Reverse transcription-polymerase chain reaction (RT-qPCR)

The total RNA was extracted from the renal tissues from the 5 groups using TRIzol reagent (Invitrogen; Thermo Fisher Scientific Inc., Waltham, MA, USA), while the RNA concentrations were determined spectrophotometrically. First-strand cDNA was synthesized using a cDNA synthesis kit (Promega Corporation, Madison, WI, USA) based on the manufacturer’s instructions. The RT-qPCR was used with the SYBR Green mix kit (Applied Biosystems; Thermo Fisher Scientific Inc.). The primers for Cytc and NGAL were designed as follows: Cytc sence, 5’-GAGCACCTTCTTTTCCTTCATCTT-3’, and antisense, 5’-TCACACACCAGCAGGTTATCATC-3’; and NGAL sence, 5’-ATGCTTCAGACCTCCCTT-3’, and antisense, 5’-CTCCACCAACTATCTCCACT-3’. GAPDH was taken as a housekeeping gene. The data were measured as a proportion of the gene to GAPDH mRNA (sense, 5’-ACAGCAACAGGGTGGTGGAC-3’, and antisense, 5’-TTTGAGGGTGCAGCGAACTT-3’). PCR was processed with 40 cycles at 92 °C for 30 s, 55 °C for 30 s, and 70 °C for 25 s, using the RT-qPCR instrument (ABI StepOnePlus; Thermo Fisher Scientific Inc.).

### 2.9. Statistical analysis

All data were presented as the mean ± SD. Statistical analyses were conducted using SPSS 17.0 (IBM Corp., Armonk, NY, USA). For comparisons of the parameters among the groups, 1-way analysis of variance was conducted after the normality tests and tests for homogeneity of variance. A difference was considered significant at P < 0.05.

## 3. Results

### 3.1. CsA ameliorated the histopathological damage in hydronephrotic kidney after perfusion

**Figure 1:**
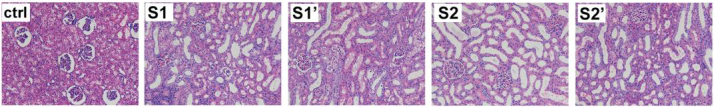
Histopathological changes in the rabbit kidneys. HE staining images of the kidney shows the swollen glomeruli and tubule cells and renal tubule dilatation (x200).

Hydronephrotic renal tissue histopathological changes in the HE staining after perfusion were observed by light microscopy. In the control group, no substantial morphological changes were observed. Renal pelvic perfusion resulted in significant damage that was represented by a swollen glomeruli and tubule cells, renal tubule dilatation, and inflammatory cell infiltration in groups S1 and S2. However, following intervention with CsA, there were fewer swollen and dilated cells in groups S1’ and S2’ than in groups S1 and S2 (Figure 1).

### 3.2. CsA ameliorated the ultramicrostructure damage in hydronephrotic kidney after perfusion

**Figure 2:**
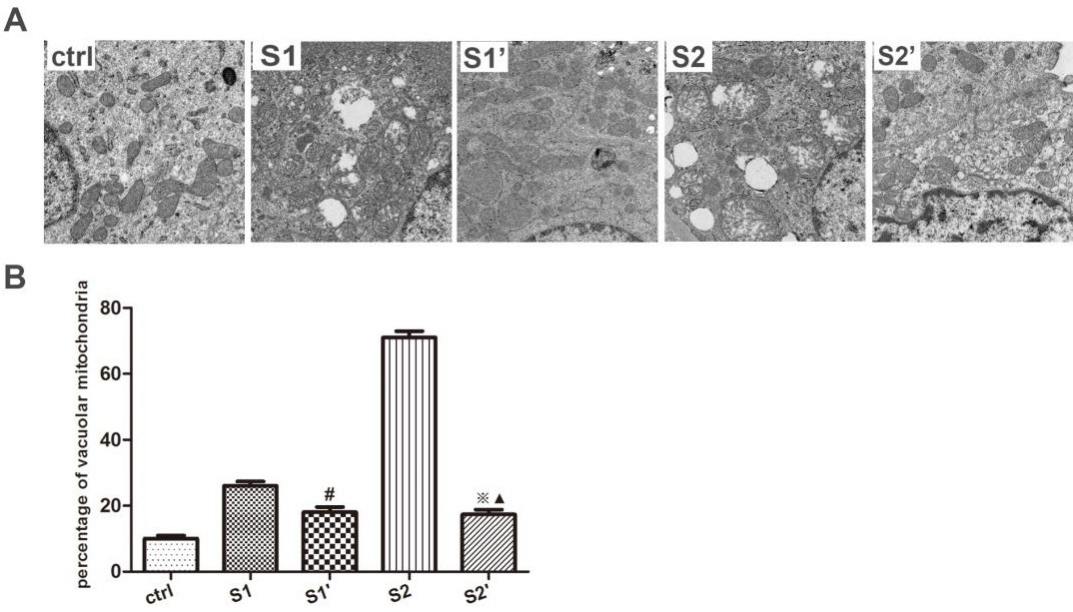
Ultrastructural changes in the mitochondria of the renal cells. (A) Transmission electron microscopy shows the vacuolar and swollen mitochondria in rabbits (×5000). (B) Quantification of the vacuolar and swollen mitochondria in rabbits. The bars represent the means ± SD; #P < 0.05 vs. S1 group,※P < 0.05 vs. S2 group,▲P > 0.05 vs. S1’ group.

Left hydronephrotic renal tissue ultrastructural changes after perfusion were observed by transmission electron microscopy. Swollen and vacuolar mitochondria of the renal tubular epithelial cells were rare in the control group. Renal pelvic perfusion resulted in significant mitochondria damage that was represented by seriously swollen mitochondria and a large number of vacuolar changes, as well as endoplasmic reticulum expansion in groups S1 and S2. However, following intervention with CsA, there were fewer swollen and vacuolar mitochondria in groups S1’ and S2’ than in groups S1 and S2 (P < 0.05). There was no significant difference between the CsA intervention groups (P > 0.05) (Figure 2).

### 3.3. CsA increased the MMP levels in hydronephrotic kidney after perfusion

**Figure 3:**
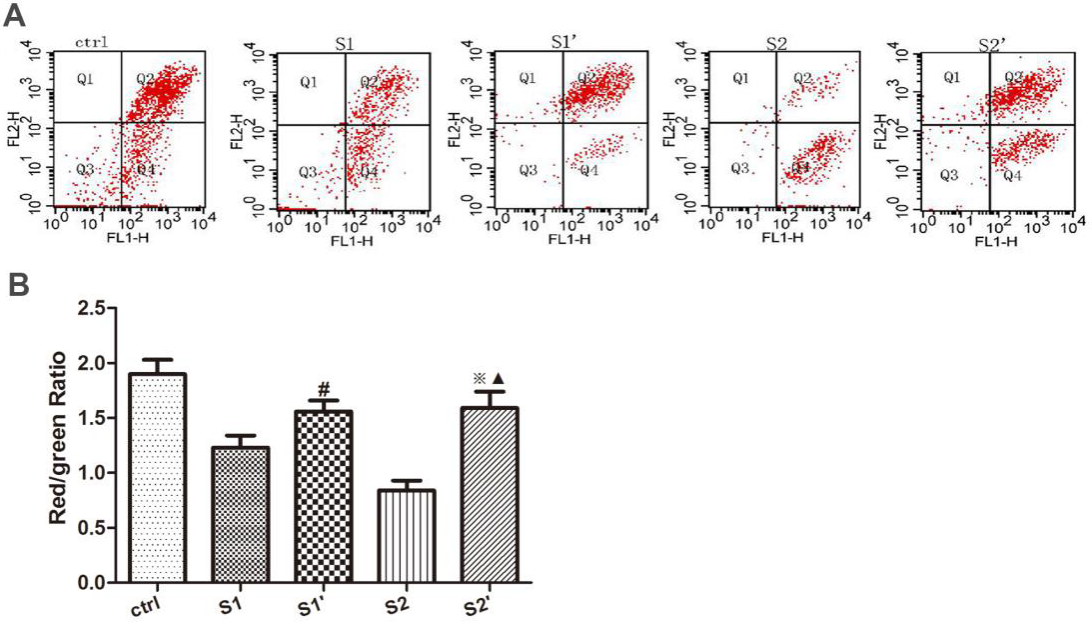
Mitochondrial membrane potential (MMP) changes in the renal cells. (A) Renal cell MMP changes analysis by flow cytometry. MMP levels are represented as the ratio of Q2 intensity to Q4 intensity. (B) Quantification of renal cell MMP in rabbits. The bars represent the means ± SD; #P < 0.05 vs. S1 group, ※P < 0.05 vs. S2 group, ▲P > 0.05 vs. S1’ group.

To determine the influence of CsA on the biomarkers of early apoptosis in the rabbit hydronephrotic kidneys after renal pelvic perfusion, MMP levels were measured using flow cytometry and JC-1 staining. The stained cells were divided into 4 subpopulations, as: section Q1, showing normal mitochondrial polarization; section Q3, showing dead cells or debris; section Q2, showing normal mitochondrial polarization and cells with mitochondrial depolarization; and section Q4, which represented mitochondrial depolarization. MMP values were presented as the proportion of section Q2 intensity to section Q4 intensity. According to the flow cytometric analysis, renal pelvic perfusion decreased the MMP levels in groups S1 and S2, but the CsA treatment effectively mediated increases in the MMP levels in groups S1’ and S2’ when compared to groups S1 and S2 (P < 0.05). There were no significant differences between the CsA intervention groups (P > 0.05) (Figure 3).

### 3.4. CsA reduced the apoptosis of hydronephrotic kidney after perfusion

**Figure 4:**
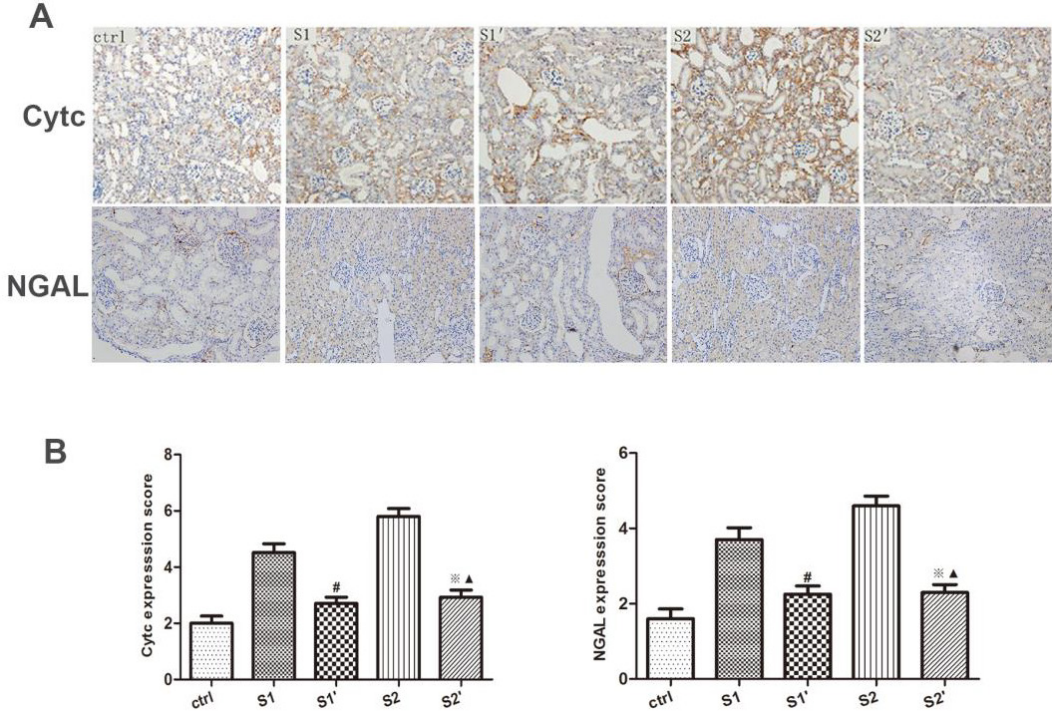
Expression of CytC and NGAL in the kidneys by immunohistochemical staining. (A) CytC and NGAL expression (×200); the brown fields in the cytoplasm represent the CytC and NGAL expression. (B) Quantification of the immunostaining scores of the CytC and NGAL expression in rabbits. The bars represent the means ± SD; #P < 0.05 vs. S1 group,※P < 0.05 vs. S2 group,▲P > 0.05 vs. S1’ group.

**Figure 5:**
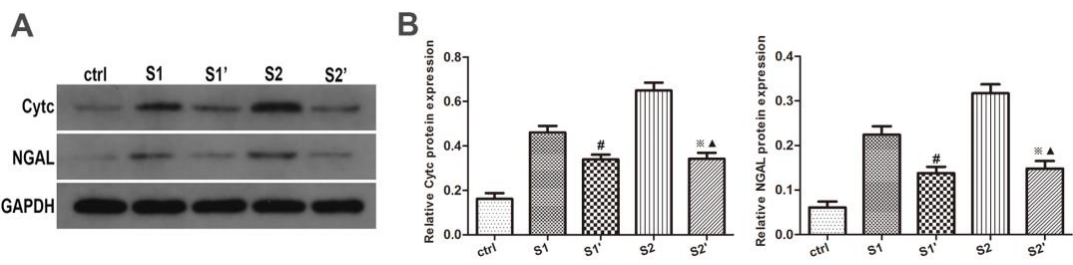
Renal protein expression changes in the Cytc and NGAL in the kidney. (A) Expression of Cytc and NGAL in the kidneys by western blotting. (B) Quantification of the Cytc and NGAL expression in rabbits. The bars represent the means ± SD; #P < 0.05 vs. S1 group, ※P < 0.05 vs. S2 group, ▲P > 0.05 vs. S1’ group.

**Figure 6:**
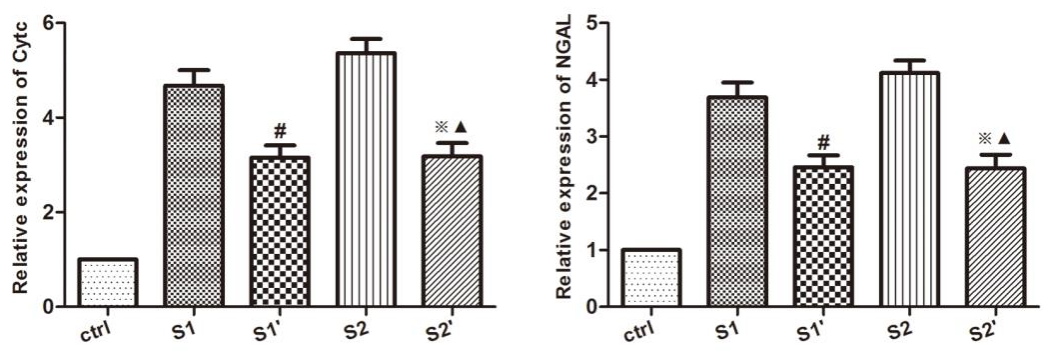
Renal mRNA expression changes in the Cytc and NGAL in the kidney. Expression of Cytc and NGAL in the kidneys by RT-qPCR. The bars represent the means ± SD; #P < 0.05 vs. S1 group, ※P < 0.05 vs. S2 group, ▲P > 0.05 vs. S1’ group

To explore the effect of CsA on apoptosis inhibition, the levels of CytC and NGAL in the rabbit hydronephrotic kidneys after renal pelvic perfusion were assessed immunohistochemically, and with Western blot analysis and RT-qPCR. The immunohistochemistry staining indicated that the rabbits in groups S1 and S2 displayed increased levels of CytC and NGAL, whereas the CsA treatment significantly lowered the levels of CytC and NGAL in groups S1’ and S2’, respectively (P < 0.05) (Figure 4). As expected, the Western blot analysis and RT qPCR showed that the CsA markedly reduced the protein and mRNA expression levels of the CytC and NGAL (Figures 5 and 6).

## 4. Discussion

Instead of traditional open surgery, flexible ureteroscope and percutaneous nephrolithotomy have been extensively applied to treat urolithiasis. These endourological surgeries, which occur in a closed cavity during the procedure, need sufficient fluid perfusion to acquire a clear operational visual field. The normal renal pelvis internal pressure is approximately 7.5 mmHg. According to a previous report [5], pyelovenous lymphatic backflow could occur with an intrapelvic pressure of more than 30 mmHg. Venous outflow obstruction can result in the formation of microthrombosis, which then affects the blood supply of the renal parenchyma. Essentially, kidney damage becomes an ischemic injury when severe hydronephrosis is subjected to a certain degree of perfusion [4,6].

In a previous experiment, it was observed that kidney damage became serious when the degree of perfusion pressure of severe hydronephrosis reached 60 mmHg [6]. Kidney damage differed depending on whether the renal pelvis was perfused with pressures of 20 or 60 mmHg. The difference was statistically significant. This is the reason why these 2 different perfusion pressures were selected in the present study. Swollen and damaged glomeruli and tubule cells, an increase in swollen mitochondria, and decreased MMP were observed in the severe hydronephrosis group when the perfusion pressure reached 60 mmHg. At the same time, a higher apoptosis rate and higher expression of apoptosis factors were observed in the severe hydronephrosis group than in the group perfused with a pressure of 20 mmHg. One mechanism by which the MMP can decrease apoptosis is the opening of the mPTP; thus, it was speculated that mitochondrial-mediated apoptosis played a dominant role in the severe hydronephrosis group. It was possible that mPTP opening and closing played a vital role in the mechanism associated with the changes in mitochondrial permeability. mPTP opening is an early sign of cell apoptosis [14]. Many methods have been applied to prevent mPTP opening and the consequent mitochondria-driven apoptosis or cell death [15–17]. In the process, the CsA has reached the certain clinical stage. Many animal experiments have confirmed that CsA has therapeutic effects on myocardial infarction [18–21] and neurogenic [22,23] ischemic injuries. A variety of research has reported that treatment with CsA during percutaneous coronary intervention induced reperfusion that was related to a smaller infarct by some measures when compared to the placebo [20]. The present study also demonstrated that CsA ameliorated the histopathological damage in hydronephrotic kidneys after perfusion.

CytC is located between the mitochondrial internal and external membranes. The increased open probability of mPTPs causes CytC to be released into the cytoplasm in the context of various stress factors, whereupon it combines with apoptosis protein activated factor (Apaf21) and adenosine triphosphate (ATP) as compound factors. These protein complexes activate the apoptotic protease cascade and mediate renal tubular epithelial cell apoptosis through caspase-dependent pathways, leading to impaired renal function [24]. In the present study, when compared with the experimental groups, the CsA intervention showed less mitochondrial vacuolization and expression of CytC, increased MMP in the renal tubular epithelial cells, and no difference between the drug intervention groups, indicating improved renal function. This showed that CsA played a protective role in maintaining the permeability and the integrity of the mitochondria. Reduced mitochondrial transmembrane potential indicated mPTP opening. As cells lose MMP, the inflow of micromolecules results in vacuolization and mitochondrial swelling, which then induces mitochondrial collapse, cell death, and a decrease in ATP synthesis [25–27]. In this experiment, CsA in rabbits was used to inhibit the decline in MMP and reduce mitochondrial membrane permeability in the renal tubular epithelial cells. Thus, the release of proapoptotic factors in the mitochondria was reduced, leading to reduced mitochondrial swelling and vacuolization. The results above implied that CsA could reduce kidney damage through an extensive mitochondrial protective effect.

NGAL is poorly expressed in normal kidney tissues. Nevertheless, it shows high expression in the proximal renal tubule during ischemic renal injury. NGAL is a sensitive and steady biomarker of renal tubule damage that is routinely used to predict kidney injury [28]. Within 3 h after kidney injury, the expression of NGAL was increased in the proximal tubule, and it was released into the plasma within 12 h. In this experiment, less NGAL expression was observed in the CsA intervention groups than in the experimental groups. While, there was no significant difference between the CsA intervention groups, these results suggested that CsA played a renoprotective role during renal pelvis perfusion through the attenuation of renal tubular damage.

CsA becomes a key component of the transplantation immunosuppressive complex by binding to cytosolic cyclophilin A. Additionally, CsA links to the cyclophilin D component of mPTP and inhibits mPTP opening. There is a general agreement that most ischemic injury of organs is associated with mitochondrial damage. Therefore, mitochondria are considered as a therapeutic target to prevent cellular injury after ischemic injury. Recent reports have shown the effects of CsA on the heart and brain [18–22]. In addition, a study found that the inhibition of cyclophilin D attenuated calcium transfer from the endoplasmic reticulum to the mitochondria in ischemia-reperfusion injury [29], as accumulated cytosolic calcium can enhance the sensitivity of the mPTP. Moreover, the protection effect of CsA in ischemia injury was also found to be associated with inhibiting the translocation of the nuclear factor of activated T cells or preventing the dephosphorylation of NOS [9,30,31]; however, the specific mechanism remains unknown and further investigation is needed.

In summary, this research provided evidence demonstrating that CsA can attenuate the renal damage of severe hydronephrosis due to renal pelvic perfusion by reducing mitochondrial injury. Drug prevention of mitochondrial injury would be a potential therapeutic approach to treat the renal pelvic perfusion-induced renal damage from severe hydronephrosis. 

## Acknowledgment

This project was supported by grants from the National Science Fund Project of China (grant no.: 81200501).
